# Effectiveness of Early Physical Therapy Rehabilitation in Patient With Juvenile Rheumatoid Arthritis

**DOI:** 10.7759/cureus.30213

**Published:** 2022-10-12

**Authors:** Sampada Meghe, Neha Chitale, Pratik Phansopkar, Aditi Joshi

**Affiliations:** 1 Department of Physiotherapy, Ravi Nair Physiotherapy College, Datta Meghe Institute of Medical Sciences, Wardha, IND; 2 Department of Musculoskeletal Physiotherapy, Ravi Nair Physiotherapy College, Datta Meghe Institute of Medical Sciences, Wardha, IND

**Keywords:** physiotherapy, pre-rehabilitation, immunosuppressive medications, strength training, juvenile rheumatoid arthritis

## Abstract

A diverse group of idiopathic inflammatory arthritis that affects children under the age of 16 and lasts six weeks or longer is known as juvenile idiopathic arthritis (JIA) or juvenile rheumatoid arthritis (JRA). It causes joint pain and morning stiffness, and the affected joints swell and become difficult to move. During flares, patients may experience flu-like symptoms such as muscle aches and fatigue. Disease-modifying antirheumatic drugs (DMARDs) are the mainstay of rheumatoid arthritis (RA) treatment. If necessary, immunosuppressive medications can be administered in stages. Nonsteroidal anti-inflammatory drugs (NSAIDs) are pain relievers for joints. Physical therapy treatment aims to reduce pain, improve joint range, correct movement patterns, strengthen weak structures, improve cardiovascular endurance, and improve patients' quality of life. We present a case of a 12-year-old boy who presented to the hospital with knee pain and an inability to walk without assistance after falling on the ground one month prior. Medical history revealed that the patient had juvenile rheumatoid arthritis, which was confirmed on investigations. The consultant physician referred the patient for pre-rehabilitation physiotherapy before corrective surgery.

## Introduction

Juvenile Rheumatoid arthritis (JRA) is an autoimmune disease in which the immune system of the body attacks the joint lining. It causes joint inflammation, which in severe cases can result in permanent joint damage and disability. Other organs that may be affected by rheumatoid arthritis include the lungs, heart, blood vessels, skin, and eyes [1]. The estimated prevalence of this disease is 48 per 100,000 in the Indian population [2]. The cause of Rheumatoid Arthritis (RA) is unknown, but certain risk factors, such as family history or other autoimmune diseases, poor dental health, and viral infections, are associated with an increased risk of developing it [3]. The first symptom of JRA is joint pain, especially in the hands and feet, followed by morning stiffness that lasts for a few minutes. RA-affected joints become swollen and difficult to move as the disease progresses [4]. Pain and swelling are frequently intermittent, with periods of increased inflammation (flares) followed by periods of relative improvement. Patients may also experience flu-like symptoms such as muscle aches and fatigue during flares [4]. The primary stay of RA treatment is disease-modifying antirheumatic drugs (DMARDs). Approximately 40% to 50% of RA patients achieve remission or have low disease activity after treatment [5]. Immunosuppressive medications can be given in stages if necessary. During flares, steroids are frequently added for brief periods to reduce inflammation [3]. Nonsteroidal anti-inflammatory drugs (NSAIDs) are medications used to alleviate joint pain. Patients with RA should be encouraged to eat well, sleep well, and exercise regularly [1]. Physical therapy treatment aims to reduce pain, improve the range of affected joints, correct movement patterns, strengthen weak structures, improve cardiovascular endurance and improve the quality of life of the patients [6].

## Case presentation

A 12-year-old male child presented to the hospital with pain in the knee and an inability to walk without support after falling on the ground one month ago. He was taken to a nearby hospital in his village, where medications were given. The pain was relieved after taking medications but reoccurred when the medications were stopped. The history revealed that the patient had rheumatoid arthritis since childhood, diagnosed at the age of five. The patient did not take any medical treatment during childhood, which resulted in an inability to run or walk fast, and due to this, the patient can only walk with support. 

Clinical findings

On general examination, the vital signs were stable. On observation, the patient was conscious and well-oriented to the surroundings. The body build was ectomorphic; the patient had genu valgus of both knees but more of the right knee, right foot plantarflexed, and swelling of small joints of both upper and lower limbs. The patient walked with the help of a walker. The pain was 6/10 on NPRS. On palpation, tenderness grade 1 was present on the anteromedial aspect of the right knee, and medial aspect of the left knee, and on proximal and distal interphalangeal joints of both hands. Active knee flexion was restricted on both the right and left sides. Crepitus was present. Muscle girth above the knee, on the thighs, and below the knee on the calf region was reduced. An X-ray confirmed displacement of the joints and decreased joint space. Blood investigations revealed elevated ESR and positive rheumatoid factor. After all the investigations, the patient was planned for knee deformity corrective surgery. Before surgery, the patient was referred to the physiotherapy department for pre-surgery rehabilitation. On assessment, the patient's range of motion was taken as mentioned in Table 1.

**Table 1:** Pre-Treatment Range of Motion

Joint	Movements	Range	Normal Range
	Flexion	80^0^	0-125^0^
Hip	Extension	20^0^	0-30^0^
	Abduction	40^0^	0-45^0^
Knee	Flexion	60^0^	0-140^0^

Therapeutic intervention 

Physiotherapy intervention was started immediately after the assessment of the patient. The patient and his parents were well-educated about the condition and the importance of pre-surgery rehabilitation. A seven-day pre-operative rehabilitation program was planned. The first goal was to decrease pain so that the child is able to move limbs without much distress. So initially, cryotherapy was given over the knee joint and ankle joint bilaterally. The patient’s family members were explained this. As the pain decreased, active mobility exercises for all the joints of the upper and lower limbs were taught to maintain mobility, starting with 10 repetitions and three sets a day. Isometrics for quadriceps and hamstrings of left and right legs for strengthening with 10 repetitions and three sets in the beginning. Dynamic quadriceps also started to strengthen the quadriceps muscle. Progressive Resistive exercises, first manually and later with therabands were taught to the patient. It was started with a yellow colored theraband, as it has the lowest resistance exercises, including strengthening of hamstrings, quadriceps, calf muscles, dorsiflexors, and plantar flexors. Hip flexion, extension, abduction, and knee flexion and extension, with theraband along with half squatting, were also taught to the patient. With the help of a walker, static exercises in standing were taught to the patient. Active flexion, an extension of the hip joint, and spot marching holding the walker. Side walking was also taught to increase the activities of the abductors. The patient and the relatives were explained the importance of these exercises before surgery and were suggested to re-visit post-surgery and consultation.

Follow-up

The range of motion of the knee joint and hip joint increased after the completion of rehabilitation (Table 2). The pain had reduced to 3/10 on NPRS, and the patient was able to perform the resisted exercise with much more ease than previously.

**Table 2:** Post-treatment Range of Motion

Joint	Movements	Range	Normal Range
Hip	Flexion	85^0^	0-125^0^
	Extension	25^0^	0-30^0^
	Abduction	43^0^	0-45^0^
Knee	Flexion	78^0^	0-140^0^

## Discussion

The patient had come in a month before with a fall history and a complaint of knee pain and inability to walk. Following a clinical evaluation, investigations, and a review of the patient's medical history, juvenile rheumatoid arthritis was diagnosed. A proper treatment protocol was developed in order to reduce pain and improve the ranges of motion in affected joints. Cryotherapy to reduce pain was given, and static and dynamic exercises were taught along with strengthening exercises with the theraband [7]. Active exercises were done while standing with the help of a walker. Patients who have good arthritis control can benefit from variable-resistance exercise programs or progressive high-intensity strength training, which can improve strength, fatigue, and pain [6]. Graded aerobic training is also beneficial [8]. Isometric exercises are prescribed for moderate disease to help maintain a functional level of muscle strength without exacerbating joint inflammation and pain [9]. Resting splints may be required at first for severely inflamed joints. Even in patients with severe inflammation, passive full range of motion exercises for all joints should be performed daily to prevent flexion contractures [6]. A systematic review of the evidence on the efficacy of interventions aimed at reducing fatigue in pediatric rheumatic condition (PRC) patients was conducted [5]. The small number of included studies, non-comparable interventions, and inconclusive results suggest that more research on the subject is needed. To reduce fatigue complaints in children and adolescents with PRCs, potential underlying biological and psychosocial mechanisms must be identified as potential treatment targets [5,8]. A study was conducted to compare the effects of two different task-oriented activity training programs on activity performance and participation in children with juvenile idiopathic arthritis. Task-oriented activity training based on video games is an alternative and feasible treatment option for children with juvenile idiopathic arthritis. Given the growing interest in virtual reality-based therapy in rehabilitation, this new method may have a widespread application in future research [10].

## Conclusions

JRA affects the bone structures and overall depletes the quality of life. Physiotherapy helps reduce pain, improve range of motion of all joints, and improves quality of life. Following one week of the treatment protocol, in this case of JRA, the patient’s pain was reduced, and he developed strength and improved range of knee and hip joint, and improved functional capacity to perform activities of daily living. This case study establishes a well-structured rehabilitation program for the patient, which is for pre-surgery rehabilitation, and also prepares the patient for post-surgery as well. The Patient showed positive outcome measures.
